# Sensor Distribution Optimization for Structural Impact Monitoring Based on NSGA-II and Wavelet Decomposition

**DOI:** 10.3390/s18124264

**Published:** 2018-12-04

**Authors:** Peng Li, Liuwei Huang, Jiachao Peng

**Affiliations:** School of Mechatronics & Vehicle Engineering, East China Jiaotong University, Nanchang 330013, China; 18270697178@163.com (L.H.); 13155825909@163.com (J.P.)

**Keywords:** structural impact monitoring, sensors distribution optimization, NSGA-II, energy analysis of wavelet band, principal component analysis

## Abstract

Optimal sensor placement is a significant task for structural health monitoring (SHM). In this paper, an SHM system is designed which can recognize the different impact location and impact degree in the composite plate. Firstly, the finite element method is used to simulate the impact, extracting numerical signals of the structure, and the wavelet decomposition is used to extract the band energy. Meanwhile, principal component analysis (PCA) is used to reduce the dimensions of the vibration signal. Following this, the non-dominated sorting genetic algorithm (NSGA-II) is used to optimize the placement of sensors. Finally, the experimental system is established, and the Product-based Neural Network is used to recognize different impact categories. Three sets of experiments are carried out to verify the optimal results. When three sensors are applied, the average accuracy of the impact recognition is 59.14%; when the number of sensors is four, the average accuracy of impact recognition is 76.95%.

## 1. Introduction

With the extensive utilization of load-carrying structures in various engineering applications, there has been increasing interest in methods for predicting and estimating the location and extent of impact damage in structures [1,2,3,4]. On the basis of recent research advances, a concept of damage diagnostics for real-time structure monitoring, namely, structural health monitoring (SHM), has been proposed. SHM aims to ensure structural safety by using information provided by the sensor network. Normally, as for a simple structure, the design of a sensor network is based on the engineers’ judgment. However, for complex structures, sensor location and the number of sensors in the network become fundamental optimization issues, which cannot be ignored. Under the premise of guaranteed performance, fewer sensors to cover an area will be considered, and the proper placing of these sensors will reduce costs.

In previous studies, sensor distribution optimization is only devoted to the location optimization of sensors, and the number of sensors is limited. This research involved in many modern heuristic algorithms such as genetic algorithm (GA) [5,6], simulated annealing (SA) [7], monkey algorithm (MA) [8], ant colony algorithm (ACO) [9] and differential evolution (DE). Kim established a novel particle swarm optimization framework to achieve robust consensus of decentralized sensors with neighbors rather than through centralized control [10]. An algorithm based on ladder diffusion and ACO is proposed to solve the power consumption and transmission routing problems in wireless sensor networks [9]. Among them, genetic algorithm has been introduced as a promising method for handling single-objective optimization, due to its better convergence, higher calculation precision, lower calculation time and higher robustness. 

However, the optimization of the sensors network requires a reduction of the number of sensors and increased monitoring accuracy. It is obviously a multi-objective optimization problem (MOOP). Inherently, sensor location and the number of sensors are two conflicting goals for the design of sensor networks. In recent years, in order to solve the multi-objective optimization problem, many researchers improved the initial heuristic algorithm [11]. Céspedes-Mota uses improved differential evolution algorithm to optimize the distribution of wireless sensor networks according to the distance arranged by sensors [12]. Deb proposes the non-dominated sorting genetic algorithm to solve multi-objective optimization problems [13]. Li also uses this method to optimize the sensors network: he uses the number of sensors and the feature difference among all impact categories as two objective functions, respectively. A set of optimal sensor networks are obtained by the NSGA-II method [14].

In this paper, the NSGA-II method is used to optimize the sensor network. The remaining part of the paper is organized as follows: Section 2 describes the problem. The two objective functions are defined as the following: (i) The number of sensors; (ii) the feature differences among different impact categories. Section 3 describes how to solve the optimization problem by using the idea of MOOP. Moreover, wavelet band energy extraction and PCA are combined to obtain the value of the objective function. The result of sensors distribution optimization by NSGA-II is described in Section 4. Meanwhile, in order to prove the superiority of genetic algorithm, a comparative study is conducted with MA. In Section 5, the performance of the proposed algorithm in optimizing sensor distribution is verified by experiments. Finally, the paper is concluded in Section 6.

## 2. Description of Problem

In this paper, the composite laminate is used as the object. The study aims to identify structural impact damage, including impact location and impact degrees, by optimizing sensor networks. The adopted material is a [0 deg/90 deg] s-glass/epoxy orthogonal anisotropic laminate. The parameters of composite laminate are shown in Table 1.

**Figure 1:**
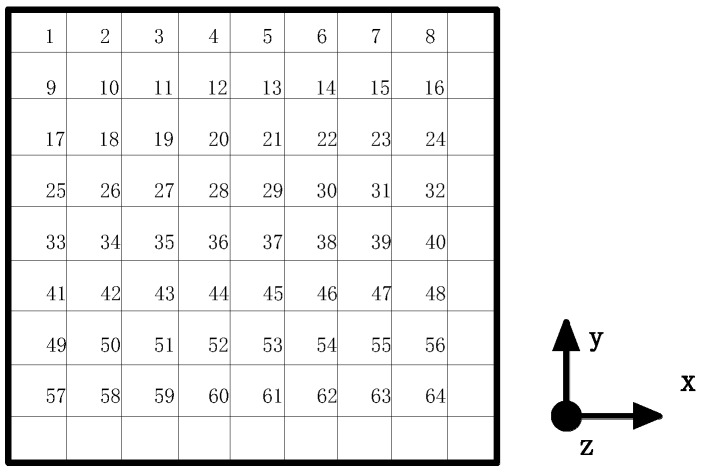
The grid division of composite.

As shown in Figure 1, the composite plate is divided into 9 × 9 grids, which includes 64 grid nodes. The impact load is applied at each node respectively. And each position is subject to two degrees impact, so there are in total 128 impact categories. The sensors are also positioned in some of these 64 nodes. Thus, there are two optimization objectives:

(1) Minimizing the number of sensors
(2) Maximizing the sensor network’s optimization performance index based on impact categories

The method of getting the objective function will be introduced in the next section.

## 3. Problem Formulation

Because there are two conflicting optimization objectives in the process of sensor network optimization, the optimization problem is a non-dominated multi-objective optimization. The optimization results will include a number of Pareto optimal solutions. Each solution is called non-dominated Pareto optimal, Pareto efficient or non-inferior. Without additional subjective preference information, all Pareto optimal solutions are considered equal. In this paper, the non-dominated sorting genetic algorithm II (NSGA-II) is used to obtain the Pareto optimal solutions of sensor networks.

### 3.1. Objective Function I

Before solving this problem, the objective function needs to be defined. Reducing the number of sensors can not only reduce the cost of monitoring system, but also accelerate the processing speed of data. The number of sensors is set as the objective function I.

In this paper, the encoding method of the sensor network is binary coding. Because the sensor has 64 alternate locations, each sensor network can be represented by 64 binary digits. When the value of the element is 0 there is no sensor in the position; when the value of the element is 1, the sensor is present in that position. So the sensor network is obtained ($\vec{S}={\left[s_{1},s_{2},\cdots ,s_{64}\right]}^{T}$). For example, if the sensor is placed in position 1, the vector $\vec{S}$ is equal to [10000000 00000000 00000000 00000000 00000000 00000000 00000000 00000000]^T^.

### 3.2. Objective Function II

#### 3.2.1. Numerical Simulation

For the purpose of improving the sensor network optimization performance index, the software ANSYS, which performs well with finite element analysis, is used to obtain the shock response of each category impact. It can provide effective data for sensor network optimization. As a rectangular structure is used in this paper, solid units are used. The composite material has a layered structure inside, so layered units are used. Solid-layered-46 is selected as the simulated entity type. Following this, the real constant is defined. The thickness of the composite plate is 15 mm, and the laminate has 10 layers, each with a thickness of 1.5 mm. The internal structure of the laminate is anisotropic orthogonal, that is, the laminate form of the laminate is (0/90) s.

**Figure 2:**
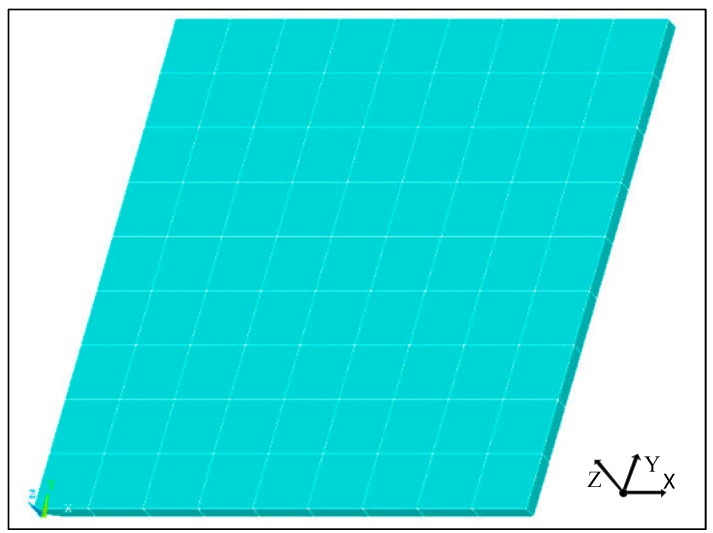
Finite element analysis model of the composite laminate.

The model is divided into 9 × 9 orthogonal distribution grids. The composite laminate is supported at the four corners, and the finite element analysis model is shown in Figure 2.

As shown in Figure 1, there are 64 nodes on the composite plate. Each node is subjected to two degrees of impact—the full-load impact and half-load impact. The impact process of full-load is divided into two steps. The first load is 30×sin(1744.4×t),0≤t≤0.0018 s and is divided into 9 sub-steps. The second load is a zero force, used in order to get the free shock response of the composite laminate. The second load includes 991 sub-steps and the total loading time is 0.1982 s. The total impact process of the simulation analysis is 0.2 s, and the load sub-steps include 1000 steps. The frequency of simulated vibration signal is 5k Hz. The half-load impact steps are similar to the whole load impact steps, the difference between them being impact strength. The half-load of the first step is 15×sin(1744.4×t),0≤t≤0.0018 s. Linear analysis is used in this paper because the impact of the experiment is elastic deformation.

**Figure 3:**
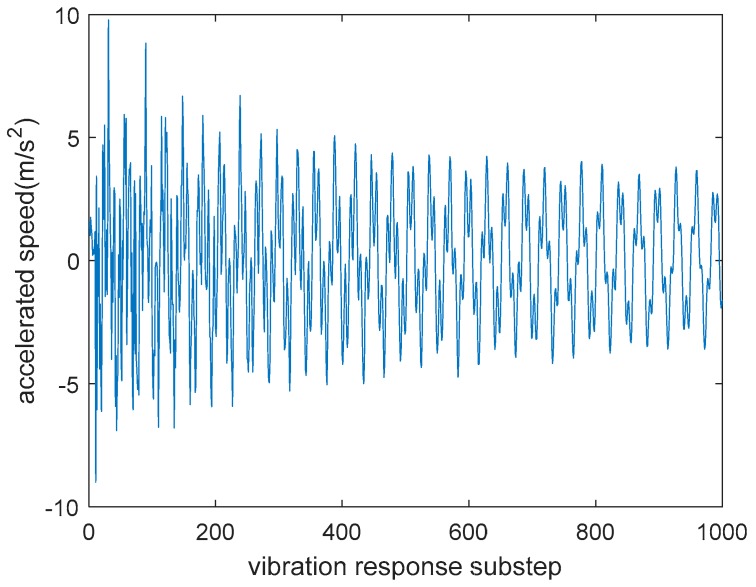
Shock response of 28th downside grid node with load acted on 28th upside grid node.

The vibration response can be obtained by ANSYS software when the structure is impacted. For example, the response of the 28th downside grid node is obtained as shown in Figure 3. The vibration response curve of the 28th downside grid node is obtained under the full-load impact to the 28th upside grid node.

Since each upside node is subject to two degrees of impact and the upside board of composite has a total 64 nodes, for the *i*th downside node it will receive a total of 128 vibration signals $\vec{M_{p}^{i}}(p=1,2,\ldots 128)$. According to the result of finite element analysis, the shock response matrix at the *i*th downside grid node $M^{i}$ is defined as

(1) $$M^{i}=\left\lceil \begin{array}{c} {\vec{M}}_{1}^{i}\\ \vdots \\ {\vec{M}}_{128}^{i} \end{array}\right\rceil =\left[\begin{array}{ccc} M_{1,1}^{i} & \cdots & M_{1,c}^{i}\\ \vdots & \ddots & \vdots \\ M_{128,1}^{i} & \cdots & M_{128,c}^{i} \end{array}\right]\left(i=1,\;2,\;\ldots ,\;64\right)$$

where *i* is the *i*th alternate sensor location and *c* is the length of simulation vibration data. As for the 64 downside grid nodes, the 64 shock response matrices are established by using the result of numerical simulation. 

#### 3.2.2. Energy Analysis of Wavelet Band

After obtaining the original simulation data, its features need to be extracted. In recent years, the wavelet transform has been introduced as a promising method in damage identification of structures due to its excellent performance in detecting signal singularity [15,16,17].

A wavelet packet transform (WPT) is an orthogonal wavelet transform (WT). It inherits the idea of short-time Fourier transform localization [18,19]. Therefore, wavelet feature extraction is often used for impact location. At the same time, the time difference localization method is also a common positioning method [20]. However, our research not only needs to identify the impact location, but also to identify the impact level. Compared with the time difference positioning method, wavelet transform can not only reflect the time difference of the signal, but also reflect the difference in signal strength. Therefore, we chose wavelet decomposition to extract features. Firstly, the principle of wavelet transform needs to be introduced. A function $\psi \in L^{2}(R)$ is called an orthonormal wavelet while it can be defined by a Hilbert basis. It is a complete orthonormal system for the Hilbert space $L^{2}(R)$ of square integrable functions. Therefore, the vibration signal can be decomposed by wavelet signal:

(2) $$M_{p}^{i}(x)=\sum_{j=1}^{N}\sum_{k\in Z}d_{k}^{j}\varphi_{jk}(x)+\sum_{k\in Z}c_{k}^{N}\varphi_{Nk}(x)$$

where, *N* is the decomposition layer, $d_{k}^{j}$ is the detail coefficient of the *j*th layer, $c_{k}^{N}$ is the approximate coefficient of the *n*th layer, and $\varphi_{Nk}(x)$ is the orthogonal scales function. According to Parseval’s theorem, the set of orthogonal functions satisfies

(3) $$\int_{R}{\left|M_{p}^{i}(x)\right|}^{2}dx=\sum_{j=1}^{N}\sum_{k\in Z}{\left|d_{k}^{j}\right|}^{2}+\sum_{k\in Z}{\left|c_{k}^{N}\right|}^{2}$$

where $E_{dN}=\sum_{j=1}^{N}\sum_{k\in Z}{\left|d_{k}^{j}\right|}^{2}$, $E_{aN}={\sum_{k\in Z}\left|c_{k}^{N}\right|}^{2}$, and $E_{dN}\;$ are known as the total energy of the detail signal of the 1 to *N* layer, $E_{aN}$ is the energy of the approximate signal of the *n*th layer. The total energy of signal $E=E_{dN}+E_{aN}$. The sum of the energy of the signals in each frequency band is consistent with the energy of the original signal, and the vibration signal in each frequency band represents the vibration characteristic information of the original signal in the frequency range. The energy expression of the *j*th layer in the vibration signal *E_j_* can be defined as

(4) $$E_{j}=\sum_{l=1}^{A}{\left|d_{l}^{j}\right|}^{2}$$

where $d_{l}^{j}$ is the decomposed signal and *A* is the number of discrete points in the corresponding time period. A set of signals are obtained which correspond to a sequence of energy from low to high frequencies $\left\{E_{j}|j=1,2,\cdots ,2^{N}\right\}$. Finally, row vector $\vec{E_{p}^{i}}$ is obtained by extracting the wavelet frequency band energy:

(5) $$E^{i}=\left\lceil \begin{array}{c} {\vec{E}}_{1}^{i}\\ \vdots \\ {\vec{E}}_{128}^{i} \end{array}\right\rceil =\left[\begin{array}{ccc} e_{1,1}^{i} & \cdots & e_{1,2^{N}}^{i}\\ \vdots & \ddots & \vdots \\ \;e_{128,1}^{i} & \cdots & e_{128,2^{N}}^{i} \end{array}\right]\;\left(i=1,\;2,\;\ldots ,\;64\right)$$

The time-frequency energy based on wavelet decomposition reflects the energy *E* of the original signal over a certain period of time. It can more fully represent the signal characteristics of the data. In order to make computation more rapid, principal component analysis (PCA) is used to reduce the dimensions of the original data.

PCA is a statistical procedure that uses an orthogonal transformation to convert a set of observations of possibly correlated variables into a set of values of linearly uncorrelated variables called principal components. The basic idea is to obtain a set of optimal unit orthogonal vectors based on the linear transformation. The sample data is then rebuilt according to the above orthogonal vector basis, in order to minimize the mean square error between reconstruction samples and original samples [21,22,23]. The orthogonality between different features is represented by the contribution rate, and then the new data are selected from the original data by setting the cumulative contribution rates $R_{m}$. Through the projection of the data from the original 2*^N^*-dimension space to *b*-dimension space (2*^N^* > *b*), namely dimensionality reduction, the new data after dimension reduction can maximally retain the information of the original data. The steps are as follows:

For the Shock response data matrix of the *i*^th^ node, the variable coefficient correlation matrix can be written as

(6) $${\left(r_{gh}\right)}^{i}=\frac{\sum_{k=1}^{128}\left(e_{kg}^{i}-\bar{x_{g}})(e_{kh}^{i}-\bar{x_{h}}\right)}{\sqrt{\sum_{k=1}^{128}{\left(e_{kg}^{i}-\bar{x_{g}}\right)}^{2}\sum_{k=1}^{128}{\left(e_{kh}^{i}-\bar{x_{h}}\right)}^{2}}}$$

where $\bar{x_{g}}=\frac{1}{128}\sum_{k=1}^{128}e_{kg}^{i}$, $\bar{x_{h}}=\frac{1}{128}\sum_{k=1}^{128}e_{kh}^{i}$, and *R^i^* = (*r_gh_*)*^i^* are the variable coefficient correlation matrix (2*^N^*, 2*^N^*). On the basis of equation $\left|R^{i}-\lambda I_{\left(2^{N},2^{N}\right)}\right|=0$, the eigenvalue of matrix *R* can be obtained $\{\lambda_{1},\lambda_{2},\ldots ,\lambda_{2^{N}}\}$. The cumulative contribution rate is described as

(7) $$R_{m}^{i}=\sum_{j=1}^{m}\lambda_{j}/\sum_{t=1}^{2^{N}}\lambda_{i}$$

where $R_{m}^{i}$ is the cumulative contribution rate, *λ* is the feature value, and *m* is the number of extracted principal component characteristics. Generally, the selection criteria of *m* need to satisfy the condition that the cumulative contribution rate is in the 85–95% range. In this study, the cumulative contribution rate is set at 95%. Then the matrix can be modeled as

(8) $$X^{i}=\left\lceil \begin{array}{c} {\vec{X}}_{1}^{i}\\ \vdots \\ {\vec{X}}_{128}^{i} \end{array}\right\rceil =\left[\begin{array}{ccc} x_{1,1}^{i} & \cdots & x_{1,b}^{i}\\ \vdots & \ddots & \vdots \\ \;x_{128,1}^{i} & \cdots & x_{128,b}^{i} \end{array}\right]\;\left(i=1,\;2,\;\ldots ,\;64\right)$$

Through this procedure, we obtained the matrix $X^{i}\left(i=1,2,\ldots ,64\right)$ which included the feature vector of all impact categories.

#### 3.2.3. Sensor Location Optimization Performance Index

The objective function II presents the sensor network optimization performance index. For all impact categories, a feature matrix *I* from a given sensor set S is shown as

(9) $$I=\left[\begin{array}{ccc} X^{s_{1}} & \cdots & X^{s_{f}} \end{array}\right]=\left[\begin{array}{ccc} {\vec{X}}_{1}^{s_{1}} & \cdots & {\vec{X}}_{1}^{s_{f}}\\ \vdots & \ddots & \vdots \\ {\vec{X}}_{128}^{s_{1}} & \cdots & {\vec{X}}_{128}^{s_{f}} \end{array}\right]$$

The feature differences among all impact categories are expressed according to the distance of the row vectors of the feature matrix *I*:

(10) $$Z=\left[\begin{array}{ccc} z_{1,1} & \cdots & z_{1,128}\\ \vdots & \ddots & \vdots \\ z_{128,1} & \cdots & z_{128,128} \end{array}\right]$$

where

(11) $$z_{u,v}=\sqrt{\left[I_{u}-I_{v}\right]\cdot {\left[I_{u}-I_{v}\right]}^{T}}\;\left(u,v=1,\;2,\;\ldots ,\;128\right)$$

Objective function II, the sensor network optimization performance index, is defined as the minimum value of the matrix element. The greater the value of the performance index, the greater the difference between the different impacts. This makes it easier to identify different impacts. In order to improve the accuracy of impact recognition, objective function II is maximized in the sensor network optimization.

## 4. Sensor Network Optimization Algorithm

Based on the previously acquired objective functions, NSGA-II is employed to optimize the sensor network. NSGA-II algorithm is proposed by Deb on the basis of NSGA [13]. Its basic process is as follows:

Step 1: Random generation of original population $\;S_{t}$. Each of these individuals $S_{t}^{g}\left(g=1,\;2,\ldots ,\;200\right)$ represent a set of sensor arrangements, and each position represents a gene. The number of sensors in each individual is the value of objective function I. Set t = 1;

Step 2: Evaluate objective function II of each sensor set. In this case, each individual corresponds to two objective function values;

Step 3: According to the value of functions, the population is divided into different non-inferior layers K_1_, K_2_, …, K*_n_*. Firstly, find the individual in K_1_. If $S_{t}^{g}\left(g=1,2,\ldots ,200\right)$ does not satisfy the following inequality:

$$f_{I}\left(S_{t}^{g}\right)\geq f_{I}\left(S_{t}^{i}\right),\;f_{\mathrm{II}}\left(S_{t}^{g}\right)<f_{\mathrm{II}}\left(S_{t}^{i}\right),\exists i=1,2,\ldots ,200$$

**Figure 4:**
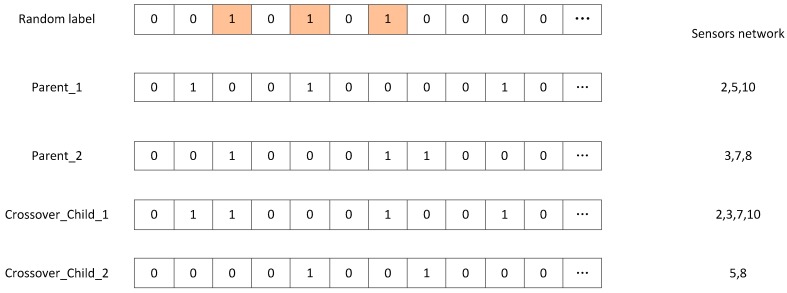
Operation of crossover.

$S_{t}^{g}\in \;$K_1_. After all individuals in K_1_ are found, these individuals are labeled, and the individuals in K_2_, …, K*_n_* are found in the same way; 

Step 4: Establish the optimization pool. Two individuals are selected randomly, and according to the non-inferior layers the better one is chosen and put into the optimization pool;

Step 5: Generating child population $S_{t}^{\prime }$ through crossover and mutation operations. Crossover operations—two individuals are randomly selected from the optimization pool and a portion of the genes in the two individuals are randomly exchanged. Figure 4 illustrates this process.

As show in the right side of Figure 4, the number of sensors between the Parent and Crossover-Child may be different. So the method could optimize different numbers of sensor networks at the same time.

**Figure 5:**
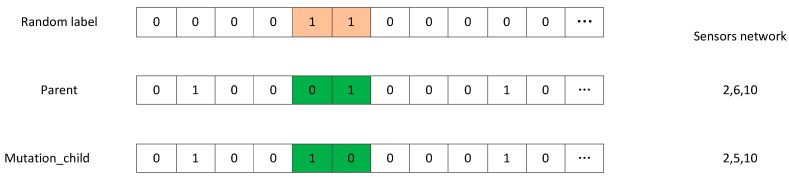
Operation of mutation.

Step 6: Mutation operations—an individual is randomly selected from the pool of preferences, and a random exchange exchanges a portion of the genes in the individual. And Figure 5 illustrates this process.

Step 7: Obtain the combined population $S_{t}^{\prime \prime }=S_{t}^{\prime }\cup^{\text{​}}S_{t}$. According to the value of objective function I and objective function II, the population is divided into different non-inferior layers K_1_, K_2_, …, K*_m_*. The highly non-inferior layers’ individuals are selected as the new population $S_{t+1}$;

Step 8: Iteration ends if the end condition is reached or t = 10,000;

Step 9: Set t = t + 1, and go back to step 3.

The parameters of the NSGA-II are shown in Table 2. 

## 5. Results and Discussion

The multi-objective optimization monkey algorithm (MOMA), composed of the monkey algorithm and NSGA-II, are used for sensor distribution optimization. The monkey algorithm (MA) is an intelligent optimization algorithm proposed by Zhao et al. [24] to solve large-scale and multi-peak optimization problems. The algorithm simulates the movement of monkeys in the process of climbing in nature, which includes climbing, looking and jumping. This algorithm includes three search processes: the climbing process is mainly used to search for the local optimal solution of the current location; the looking process mainly searches the neighborhood for better solutions than the current position in order to accelerate the search process of the optimal solution; the jump process is to search other areas to avoid the search process in the local area. In this paper, the concept of deep climbing is introduced on the basis of the classical monkey algorithm, which increases the range of optimal solutions and accelerates the convergence rate of the algorithm [25]. As the total number of positions of the sensor is 64, the fast distance is 64. In this paper, the climb process, watch–jump process and deep climb process thresholds are 8, 64 and 64, respectively.

**Figure 6:**
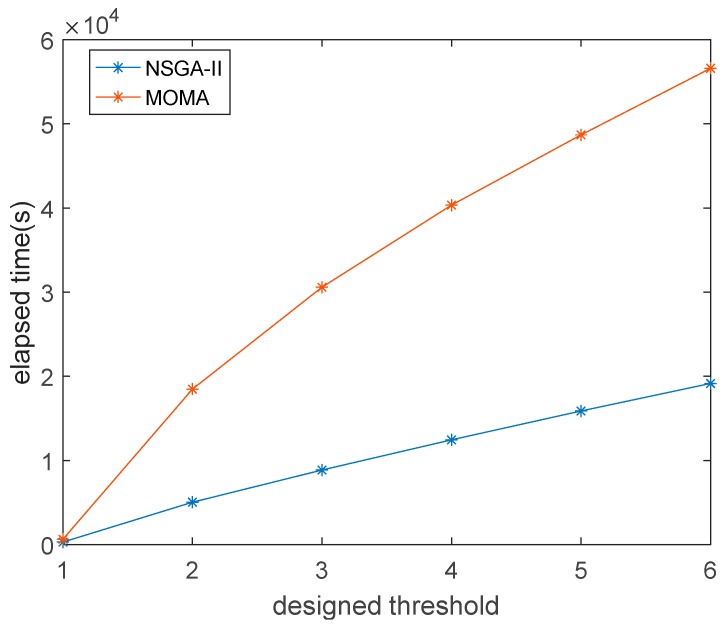
Elapsed time of (non-dominated sorting genetic algorithm) NSGA-II and multi-objective optimization monkey algorithm (MOMA) for the different designed thresholds of sensor number.

The sensor network optimization results of NSGA-II and MOMA are obtained as shown in Table 3. From Table 3, it is observed that for the different designed thresholds of sensor number, all sensor sets of low threshold are included in sensor sets of high threshold. The NSGA-II and MOMA algorithms get the same optimal sensors network. This proved that the arrangement of the sensor network is optimal. The iteration times of the two algorithms are shown in Figure 6. Since the two algorithms get the same optimal solution, the convergence rate becomes the main index to decide which algorithm is better. NSGA-II is better than the MOMA in terms of iteration time.

## 6. Method Evaluation

In order to verify the accuracy of impact recognition by sensor networks, an experimental system is designed. In this section, the experimental equipment and classification algorithm are introduced and experimental results are presented.

### 6.1. Experimental Setup

In this experiment, the identification includes impact degrees and impact locations. We suppose a rubber ball with a diameter of 20 mm dropped from 250 mm is considered a full-load shock, and the height of 125 mm freely dropped is seen as a half-load shock. At the same time, the difference of recognition accuracy and computation time between two, three and four sensors are also considered in the experiment.

**Figure 7:**
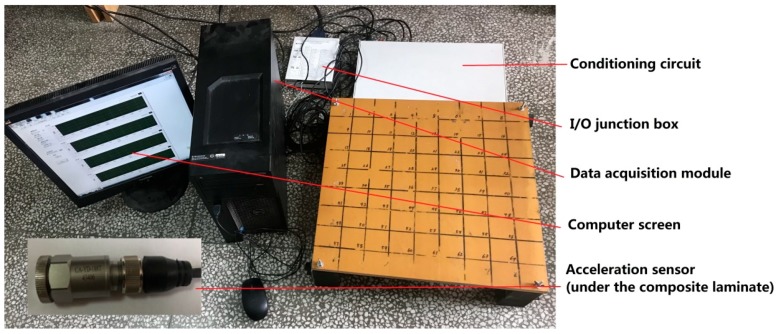
Experimental system.

The experimental system is shown in Figure 7. Firstly, the vibration signal is collected by the acceleration sensors. Secondly, the signal is introduced into the conditioning circuit to amplify the signal. Thirdly, the signal is imported into the data acquisition module. The parameters of the experimental installations are shown in Table 4.

### 6.2. Localization Methodology

In the experiment, 100 samples are taken from each kind of full-load and half-load impact. At the same time, the experiments are carried out when the number of sensors network is 2, 3 and 4, respectively. Taking the number of sensors 4 as an example, the calculation process of impact category recognition is introduced.

Step 1: Dividing the original data. Fifty groups are randomly selected as training samples, and the other 50 groups are selected as test samples. Each category’s training sample of a sensor is selected according to the similarity of vibration waveforms. If the average similarity is less than 0.5, this group of data is deleted. From the rest of the data, 30 groups are randomly selected as training samples, so a training sample matrix is obtained.

Step 2: Getting the training sample feature matrix. In the first place, energy analysis of wavelet band is used to extract features from vibration data. Then, principal component analysis (PCA) is used to reduce the dimensions of the training sample matrix. The cumulative contribution rate is 95%. Following this, the data of the 4 sensors are merged together to obtain the feature matrix $X_{q}^{p}\left(p=1,\;2,\;\ldots ,\;128,\;q=1,\;2,\;\ldots ,\;30\right)$.

(12) $$X^{p}=\left\lceil \begin{array}{c} {\vec{X}}_{1}^{p}\\ \vdots \\ {\vec{X}}_{30}^{p} \end{array}\right\rceil =\left[\begin{array}{ccc} x_{1,1}^{p} & \cdots & x_{1,m\times 4}^{p}\\ \vdots & \ddots & \vdots \\ \;x_{30,1}^{p} & \cdots & x_{30,m\times 4}^{p} \end{array}\right]\;\left(p=1,\;2,\;\ldots ,\;128\right)$$

Step 3: Selecting test samples. The average similarity between each test sample of each set of sensors and the shock response of each group’s 30 training samples is calculated, respectively. If the similarity between all of the 128 impact classes is less than 0.5, the data of this shock are deleted. Afterwards, 20 groups from qualifying data are selected as test samples.

Step 4: Obtaining the test sample feature matrix. Test samples of the 4 sensors are multiplied by 4 transformation matrixes and the first *m* data are saved just like the training sample. Then, the matrices of the 4 sensors are combined into the same column, and the characteristics of the test samples for the *p*^th^ vibration data $B_{t}^{p}\left(p=1,\;2,\;\ldots ,\;128,\;t=1,\;2,\;\ldots ,\;20\right)$ can be expressed as

(13) $$B^{p}=\left\lceil \begin{array}{c} {\vec{B}}_{1}^{p}\\ \vdots \\ {\vec{B}}_{20}^{p} \end{array}\right\rceil =\left[\begin{array}{ccc} b_{1,1}^{p} & \cdots & b_{1,m\times 4}^{p}\\ \vdots & \ddots & \vdots \\ \;b_{20,1}^{p} & \cdots & b_{20,m\times 4}^{p} \end{array}\right]\;\left(p=1,\;2,\;\ldots ,\;128\right)$$

Step 5: Identification of impact category by Product-based Neural Network (PNN). There are many algorithms for solving classification problems, among which PNN is a neural network commonly used in pattern classification [26]. Its training time is short and its classification accuracy is high. No matter how complex the classification problem is, as long as there are enough training data, the optimal solution under the Bayes criterion can be obtained. Therefore, this paper uses PNN algorithm for classification. 

When inputting a vector $\vec{B_{t}^{p}}\left(p=1,\;2,\;\ldots ,\;128,\;t=1,\;2,\;\ldots ,\;20\right)$, the pattern layer computes the distance between the input vector and the training vectors $\;\vec{X_{q}^{p}}\left(p=1,\;2,\;\ldots ,\;128,\;q=1,\;2,\;\ldots ,\;30\right)$. The summation layer sums the contribution for each class of inputs and output a probability value $f_{p}\left(p=1,\;2,\;\ldots ,\;128\right)$.

**Figure 8:**
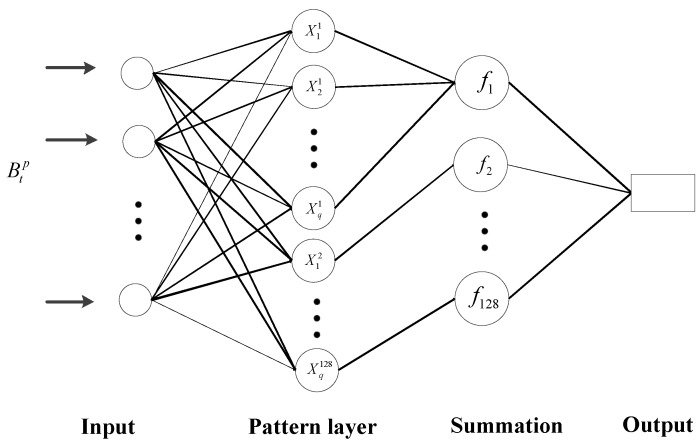
Probabilistic neural network structure for impact recognition.

Figure 8 shows the basic design of a PNN used for impact recognition. The feature vector $\vec{B_{q}^{p}}$ passes from the input layer through the pattern layer to the output layer. The neurons in the pattern layer enable mapping of the nonlinearity relations between the input and output values, which gives PNN models a better performance over others. The summation $f_{p}$ is expressed as

(14) $$f_{p}(B_{t}^{p})=\frac{1}{a{\left(2\pi \right)}^{m\times 4/2\sigma^{m\times 4}}}\sum_{q=1}^{a}\exp(-\sum_{w=1}^{m\times 4}\frac{{\left(b_{tw}^{p}-x_{qw}\right)}^{2}}{2\sigma^{2}})$$

where *a* is the training samples number of each category, *σ* represents the smoothness parameter, and the value of the smoothed parameter is 0.15. $b_{tw}$ represents the *w*^th^ data of the *t*^th^ neuron of each sample. The summation layer has 30 neurons in each, with a total 128 categories. The output layer compares the votes for each target predict accumulated in the summing layer. The target category is predicted to the largest vote.

### 6.3. Experimental Results and Discussion

Next, in order to confirm the effectiveness of our optimization results, four sets of sensor networks are selected for impact experiments. They are the first non-inferior layer arrangement of 2, 3, and 4 sensors, respectively. And the four sensors in the second non-inferior layer are also arrangement.We analyze the difference in impact identification between the different number of sensors and sensor locations. The impact recognition accuracy obtained by the above method is shown in Figure 7.

**Figure 9:**
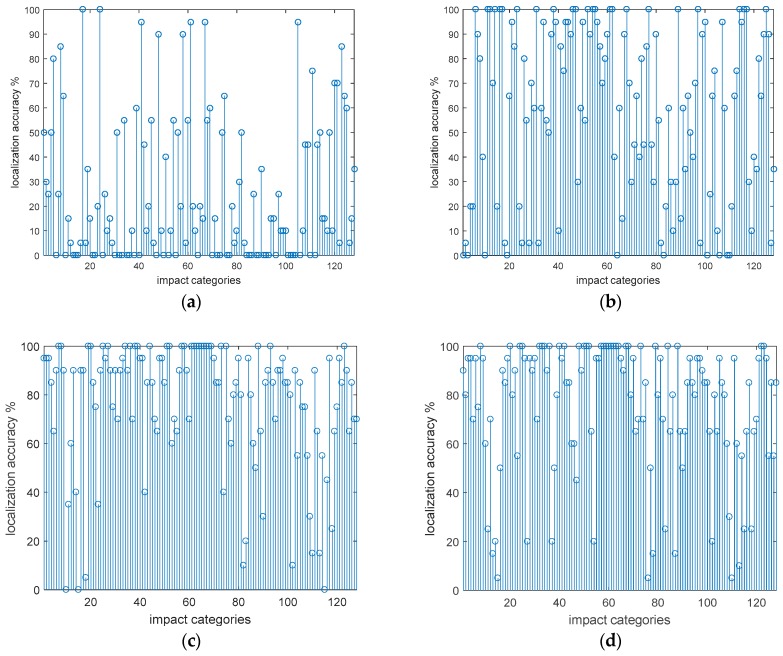
(**a**) The positioning accuracy when installing two sensors; (**b**) the positioning accuracy when installing there sensors; (**c**) the positioning accuracy when installing four sensors; (**d**) the positioning accuracy when installing four sensors, which are second non-inferior layer (10, 15, 35, 38).

As shown in Figure 9, each figure includes 128-type impact recognition accuracy. The first 64 impact categories are half-load shocks and the last 64 are full-load shocks. From Figure 9, the recognition accuracy gradually increases with the number of sensors. Recognition accuracy of the half-load is slightly higher than recognition accuracy of the full-load. Table 5 gives detailed data.

## 7. Conclusions

This paper proposes an optimization program of sensor networks for impact identification of composite laminates. The optimization objective includes the optimization of the number of sensors and the sensor network optimization performance index. The number of sensors is defined as objective function I, and the sensor location optimization performance index is defined as objective function II. In order to obtain objective function II, the finite element, energy analysis of wavelet band and PCA methods are used to data processing. Moreover, an experimental system was established to verify whether the impact recognition results were consistent with the assumptions. The final recognition accuracy is obtained using the above mentioned experimental system, and the conclusions are showing as follows:

(a) Comparing NSGA-II and MOMA, the two algorithms can get the same result. However, NSGA-II is better than the MOMA in terms of iteration time, and the gap will become more pronounced with increasing numbers of sensors;
(b) When the number of sensors in the sensor network is 2, 3, and 4, the average recognition accuracy is 25.27%, 59.14% and 76.95%, respectively. The results show that as the number of sensors increases, higher recognition accuracy is obtained;
(c) When there are four sensors, the identification accuracy of the first non-inferior sensor network is 76.95%, and the recognition accuracy of the second non-inferior sensor network is 75.55%. Results show that when the number of sensors is constant, the accuracy of impact recognition will be correlated with the sensor network optimization performance index. The experiment shows that the optimized sensors network can achieve the best impact recognition accuracy;
(d) When there are two sensors in the experiment, the average of full-load impact and half-load impact are 23.67% and 26.88%, respectively; with three sensors, the average of full-load impact and half-load impact are 52.97% and 65.31%, respectively; and with four sensors in the experiment, the average of full-load impact and half-load impact are 72.19% and 81.72%, respectively. The results show that the accuracy of the half-load is slightly higher than full-load under the same conditions;
(e) The calculation time of the experimental results shows that when the number of sensors increases, the recognition accuracy increases. However, computing time also increases. Therefore, in real application, it is necessary to select the appropriate number of sensors according to the real-time requirements.

## Figures and Tables

**Table 1:** Parameters of composite laminate.

Equipment	Parameter
thickness and area	15 mm × 500 mm × 500 mm
elastic modulus	E_z_ = 7.2 GPa, E_x_ = E_y_ = 6.9 GPa
Poisson ratio	V_x__z_ = V_y__z_ = 0.29, V_xy_ = 0.28
shear elasticity	G_xz_ = G_y__z_ = 7.6 GPa, G_xy_ = 4.4GPa
density	2100 Kg/m^3^

**Table 2:** Parameter set of genetic algorithm (GA).

Parameter	Numeric
initial population size	min(n × 64,200)
crossover probability	0.5
mutation probability	0.16
off-springs population size after mutation operation and crossover operation	min(n × 64 × 1.5300)
termination condition	min(n × 64,200) (selection according to crowding distance)

**Table 3:** Sensor sets of Pareto solutions with the different designed thresholds of sensor number.

Designed Threshold	Solution No.	Sensor No. (NSGA-II)						Sensor No. (MOMA)					
6	6	4	5	35	36	37	38	4	5	35	36	37	38
	5	18	27	28	30	55		18	27	28	30	55	
	4	27	30	50	55			27	30	50	55		
	3	11	25	29				11	25	29			
	2	28	30					28	30				
	1	25						25					
3	3	11	25	29				11	25	29			
	2	28	30					28	30				
	1	25						25					
1	1	25						25					

**Table 4:** Experimental installation parameters.

Equipment	Model Number	Parameter
acceleration sensors	CA-YD-188T	with a range of −10 g to 10 g, sensitivity is 500 mV/g, frequency response is 0.6~5000
conditioning circuit	YE3826A	12 channels, with a gain of 10, the electric current output is 4 mA
I/O junction box	NI SCB-68A	16 channel analog input channel, custom cable connector kits and mounting accessories
data acquisition module	NI PCI-6251	16 analog inputs at 16 bits, 1.25 MS/s (1 MS/s scanning), Up to 4 analog outputs at 16 bits, 2.8 MS/s (2 μs full-scale settling), Analog and digital triggering, Two 32-bit, 80 MHz counter/timers

**Table 5:** Different sensors network impact accuracy.

Sensors Network	Average Accuracy	Half-Load Impact Average Accuracy	Full-Load Impact Average Accuracy	Computation Time
(28, 30)	25.27%	26.88%	23.67%	6.01 ms
(11, 25, 29)	59.14%	65.31%	52.97%	8.36 ms
(27, 30, 50, 55)	76.95%	81.72%	72.19%	11.33 ms
(10, 15, 35, 38)	75.55%	80.23%	70.86%	11.33 ms
